# Creative Polymathy and the COVID-19 Crisis

**DOI:** 10.3389/fpsyg.2020.601508

**Published:** 2020-12-15

**Authors:** Michael Espindola Araki, Angela J. Cotellessa

**Affiliations:** ^1^Forcht Center for Entrepreneurship, College of Business, University of Louisville, Louisville, KY, United States; ^2^Graduate School of Education and Human Development, The George Washington University, Washington, DC, United States

**Keywords:** polymathy, polymath, COVID-19 crisis, interdisciplinarity, creativity and innovation, coronavirus, individual differences, theoretical framework

## Abstract

It is increasingly argued that polymathy—vocational and avocational pursuits in multiple domains—is deeply associated with creativity and innovation, and that its development enables the creation of important bridges between otherwise fragmented, dispersed sets of knowledge. Nevertheless, the dominant culture in both industry and academia is still that of narrow specialization. In this paper, we argue that in the context of COVID-19 crisis, with its wicked and transdisciplinary nature, the disciplinary approach of specialization is ill-suited to solve our increasingly complex problems, and that polymathic thinking can be a crucial asset in this regard. Drawing on different literature strands, we first examine the interplay between polymathy and other well-developed constructs in personality and temperament research. We then advance theoretical predictions regarding the relationship between trait polymathy and resilience in the COVID-19 crisis. After that, we discuss learnable strategies that can be used in complex, uncertain and adverse situations, which are associated with development of a more polymathic (broader, deeper and more integrated) set of knowledge. Later, we discuss how it may be possible to better capitalize on the key features of polymathic thinking at the societal level. Finally, we conclude with a reflection on the adequacy of our current institutions for dealing with complex problems, and we underscore the crucial role of polymathic thinking in an increasingly complex and interrelated world.

## Introduction

In late 2019, COVID-19 appeared, being considered a “once in a lifetime” pandemic, changing our world to the extent that many people may see life as broken up into two halves: before the pandemic, and after it. Clearly, this experience has been extremely impactful: not only has the COVID-19 pandemic been a global health crisis but it has also affected the way we conduct our lives, consume, work, and learn. Also, with its drastic impact, the crisis has brought into public attention crucial questions. What can we learn from this experience? What could have been done better? Further, what can this global pandemic teach us about how to prepare for and react to future wicked challenges that can pose a threat to our species?

We posit that one way to be better prepared for future wicked and complex challenges is to develop a broader, bolder, and cross-disciplinary kind of inquiry: a polymathic perspective. This perspective is based on the research on polymaths—thinkers that have navigated seamlessly across different oceans of knowledge and espouse perfectly the kind of broad, profound, and integrative thinking that has become crucial today (Araki, 2020). Previous research has found that polymathy may be a crucial element for creativity, especially in contexts that are novel, transdisciplinary, or which require a shift in perspective (Sriraman and Søndergaard, 2009; Gombrich, 2016; Root-Bernstein and Root-Bernstein, 2020b). However, despite the general claim that creativity and innovation are important and despite the role of polymathy for creativity and innovation to flourish (Simonton and Cassandro, 2010; Root-Bernstein et al., 2019), the dominant culture in both the industry and academia is that of specialization. To be successful as a professional, one is expected to focus very narrowly, avoid giving “mixed messages” regarding one’s expertise, and often advised to split one’s interests and passions between those that are “vocational”—and deserve one’s true attention—and those that are “avocational,” and should not take precious time away from one’s main specialty (see also Root-Bernstein and Root-Bernstein, 2004; Root-Bernstein, 2009; Araki, 2018). In this paper, we contend that not only this paradigm is hurtful for polymathic people but also it is ill-suited to deal with the wicked, transdisciplinary kind of problems that events such as the COVID-19 crisis bring.

This paper contributes to the literature with an in-depth examination on the role of polymathy and polymathic thinking as a tool to prepare for and solve serious problems that are not amenable to the traditional disciplinary approach. Additionally, we explore the relationship of polymathy with resilience in helping individuals and organizations adapt to the current and upcoming changes posed by the COVID-19 pandemic, and how polymathy can help solve “small” and big challenges arising from the COVID-19 crisis. In sum, we articulate polymathy with both the big problems that humankind faces and the smaller—albeit very consequential at the individual level—problems that most people and professionals face in their daily lives. For that matter, the COVID-19 crisis represents a unique case of a major disruption, occurring in the lives of millions of people, practically simultaneously, worldwide.

The remainder of the paper is organized as follows. Given the nascent nature of polymathy studies, we begin by reviewing the conceptual domain of polymathy and we then discuss relevant theories and previous research on the construct. Next, we focus on the construct of trait polymathy and discuss theoretical predictions regarding the relationship between trait polymathy and resilience in the COVID-19 crisis. Additionally, we discuss the interplay between trait polymathy and personality and temperament characteristics. Then, we shift the focus to the learnable strategies associated with polymathy and we advance the idea of the push-type polymathy; i.e., when because of reasons unrelated to their personal attributes, people are put in a position that “pushes” them toward the development of a more polymathic (broader, deeper, and more integrated) set of knowledge and skills. After that, we discuss the special role of polymathy for problems that have a “wicked” transdisciplinary nature, such as the COVID-19 crisis, and we discuss possible strategies to better capitalize on the key features of polymathic thinking. Finally, we conclude with a reflection on the adequacy of our current institutions for dealing with complex problems, and with a discussion regarding the role of polymathic thinking in an increasingly complex and interrelated world.

## The Conceptual Domain of Polymathy

Polymathy represents an idea almost as old as civilization, dating back to about 2,500 years ago when philosophers in Ancient Greece combined the prefix *poly-* (many or much) with the root of *mathemata* (things learned through experience, autodidacticism, or through classes) (Liddell and Scott, 1896). Importantly, there has been no consensus regarding its definition, its conceptual domain and its boundary conditions (Root-Bernstein and Root-Bernstein, 2020b). Polymathy has been associated with personal values and worldviews (Sriraman, 2009), ability (Kaplan and Kaufman, 2017, p. 9; Shavinina, 2013), personality traits (Araki, 2018), acquired knowledge and skills (Marrou, 1956), creativity (Beghetto and Kaufman, 2009), and eminence (Burke, 2020, 2010). For instance, researchers focusing on knowledge acquisition in different domains tend to define polymathy in terms of breadth and depth of one’s acquired knowledge (e.g., “knowledge and skills in a wide range of disciplines,” Gombrich, 2016, p. 75; or “knowledge extending over every kind of specialized study,” Marrou, 1956, p. 55). Alternatively, researchers focusing on creative processes and outcomes tend to define polymathy in terms of creative magnitude (e.g., little-c, Pro-c, Big-C; Beghetto and Kaufman, 2009) in multiple domains. This polysemy regarding the term polymathy suggests that, instead of a unitary construct, polymathy has been used as an umbrella term for a number of different but related constructs.

**FIGURE 1:**
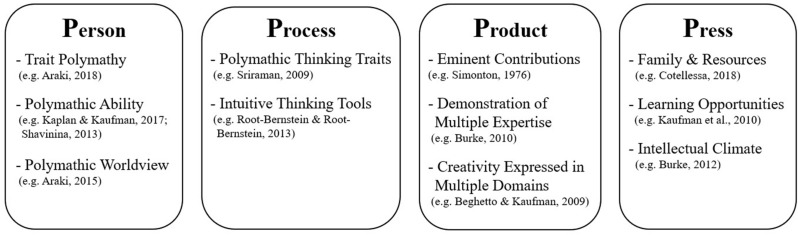
The four Ps in polymathy research.

To better comprehend and organize a new construct such as polymathy, we draw on the lessons learned by creativity researchers in the task of defining the also multifaceted construct of creativity. Particularly, creativity researchers found that it was useful to analyze the phenomenon through four distinct perspectives: the person, process, product, and press (Rhodes, 1961). This approach, known as the 4Ps of creativity, can be equally useful to organize the different perspectives on polymathy (Figure 1). The *person* perspective pertains to the abilities, traits and fluctuating state characteristics of the person; it may involve observable evidence as well as more inferred features, including cognitive styles, and affective and motivational patterns, such as intentions, attitudes, and values (Richards, 1999). Examples of polymathy-related constructs that fall within this perspective are trait polymathy and polymathic abilities (Araki, 2018). The *process* perspective includes particular ways of thinking, feeling and experiencing the world, and behaviors that are related to the generation of polymathic outcomes. Of particular interest are the processes that may be relatively unique, or necessary—even if not sufficient—to the generation of polymathic outcomes (cf. Richards, 1999). Examples of constructs that fall within this category are the thinking tools of polymaths (Root-Bernstein and Root-Bernstein, 2013) and polymathy as a thinking trait (Sriraman, 2009). The *product* perspective pertains to the results or outcomes of polymathic efforts, it can involve outcomes as diverse as a concrete product, a repertoire of performances, or a set of political ideas to be communicated. An example of this perspective is to assess polymathy as achieved eminence in more than one domain or subdomain (e.g. White, 1931; Simonton, 1976). Finally, the *press* perspective analyzes the environmental conditions that favor (or disfavor) the development of polymathy. An example of this perspective if found in Cotellessa (2018) when the author examines how particular conditions (e.g., the availability of resources and family encouragement) have facilitated or restrained the development of polymathic people.

Despite the non-homogeneous conceptualizations and definitions of polymathy, studies have identified distinguishing characteristics about the phenomenon, including traits and behaviors that are either closely related to or arise from polymathy-based constructs. Sriraman (2009) found that mathematics educators with more polymathic traits behave differently from those with less or no polymathic traits while dealing with difficult problems. The differences include that polymathic people use frequent shifts in perspective, they think more with analogies, and they have a tendency to think nepistemologically; i.e., they question the paradigms, worldviews, methods and heuristics commonly used in a domain. Cotellessa (2018) found that individuals with accomplishments in either arts or sciences and with significant expertise in the other area face unique contradictions, challenges and benefits, that are particular of their polymathic profile. Relatedly, Root-Bernstein et al. (1993, 1995, 2008); Root-Bernstein and Root-Bernstein (2020a) found that eminent creators and leaders (Nobel prize laurates) tend to be unusually polymathic—i.e., they put a significant amount of time and effort in activities that are outside their common professional range—whereas their less successful counterparts tend not to engage so much in interests outside their common disciplinary boundaries.

### The General Construct of Polymathy

Although earlier research has brought several insights into the phenomenon of polymathy, the existence of a multitude of polymathy-based constructs may lead to lack of clarity and to confusions that can stifle theoretical and empirical progress in polymathy studies. Thus, it is important to develop our understanding about what constitutes polymathy as a general construct (see also Araki and Pires, 2019; Root-Bernstein and Root-Bernstein, 2020b).

We argue that at the crux of the polymathy construct are the acts of acquisition and effectuation of knowledge, and the processes and traits that may underlie these acts. As seen, it has led to different objects being qualified as polymathic, such as one’s knowledge and skills, creative achievements, and thinking processes and dispositions. Thus, for a more unified understanding, special attention must be given to the criteria utilized to qualify any of these objects as polymathic.

Araki (2018, 2015) found that two criteria are often used to assess whether one’s products qualify as polymathic: the criteria of “depth” and “breadth.” Additionally, especially under the person or process perspectives, another criterion is needed: that of “integration.” Thus, the criterion space for polymathy is only complete when the elements of depth, breadth and integration are considered in conjunction. Depth often refers to the vertical accumulation of knowledge—knowledge which, under the product perspective, eventually leads to demonstrable expertise or creative products. For the person and process perspectives, the criterion of depth should include the processes, traits and dispositions that are associated with the vertical accumulation of knowledge, as well as the acquisition and effectuation of expertise. Breadth often refers to the latitude of knowledge and to its diversity; it involves learnings in unrelated domains and a repertoire that contain less typical combinations of knowledges and experiences. For the person and process perspectives, this criterion should include the processes, traits and dispositions associated with a wider latitude and diversity of knowledge. Finally, integration is the only criterion that becomes more explicit under the person or process perspective, and it refers to the disposition to or the effectuation of connections between ideas, methods, heuristics, principles, techniques, materials, styles, and frameworks that are considered to belong to distinct domains. The idea of integration also goes beyond the connections that happen at the ideational level so as to include the development of a personal, subjective psychological integration (see “synergistic networks of enterprise,” Gruber, 1989; “integrated activity sets,” Dewey, 1934; and “correlative talents,” Root-Bernstein, 1989). Furthermore, integration has been associated with the Humboldtian “most general, most animated, and most unrestrained” interplay between the person and their environment through the development of an informed, reflective and transformative posture in the world (Humboldt, 1794/2012; see also *Bildung*; Peukert, 2002). This conceptualization of integration also draws on the psychological concepts of “integrated personality” (Young, 1923), “propriative striving” (Allport, 1955), and “self-actualization” (Maslow, 1965), thus it spans all aspects of polymathy related to how well a person connects, synthesizes, balances, or integrates their dispositions, their knowledge and their productive pursuits (see also Kaufman, 2020).

The presented view of polymathy is consistent with the recent definition advanced by Root-Bernstein and Root-Bernstein (2020b), p. 375) of polymathy as “active engagement in multiple interests or endeavors that draw upon or synthesize vocations and/or avocations, simultaneously or serially, across the lifespan.” In this definition, all of the three previously mentioned criteria are represented. First, active engagement presupposes endeavors that are not superficial, differentiating the polymath from the dilettante, and highlighting again the role of *depth*. Second, the multiplicity of interests and endeavors represent the criterion of *breadth*. Finally, the capacity to draw upon or synthesize vocations and/or avocations into productive rather than unproductive ends underscores the importance of *integration*.

This greater clarity regarding the components that pertain to the general construct of polymathy is also useful to compare polymathy with similar constructs. For example, the construct of the T-shaped professional, like polymathy, presumes depth of expertise. However, the assumptions regarding breadth are somewhat different. While for the T-shaped professional, breadth represents “a shallow, admittedly superficial awareness of a broad range of different fields” (Sawyer, 2013, p. 63), for general polymathy, breadth must go beyond superficial awareness, dilettancy, or passive understanding, and it must inform in a more integrated and profound manner how one can effectuate their wide-raging knowledge productively (Root-Bernstein and Root-Bernstein, 2020a). Finally, the aspect of integration is also assumed within the T-shaped professional, since this professional is expected to form new connections between their main expertise (the vertical bar of the T) and potentially useful concepts and discoveries in other areas (the horizontal bar of the T). The polymathy construct not only helps to make this integration aspect explicit but also extends the idea of integration beyond the type of connection that occurs at the level of ideas to include the type of integration that occurs at the personality level, as well as between the individual and society.

### The Developmental Model of Polymathy

In a previous effort to identify the boundary conditions and to integrate the main constructs associated with polymathy, Araki (2018) proposed the developmental model of polymathy. The model posits that the pursuit of polymathy can be understood as a lifelong “project,” whereby the many different facets of a person’s psyche and behavior can be integrated through the lens of a “polymathic worldview.” The zenith of the polymathic life project is to become a wholly formed individual, who is able to produce creative contributions and has developed a consciousness highly capable of reflective awareness of the self, of their relationship to others, and of their relationship to the world. This view draws particularly on the Humboldtian ideal of self-formation; the achievement of “as much substance as possible for the concept of humanity in our person” (Humboldt, 1794/2012, p. 58).

**FIGURE 2:**
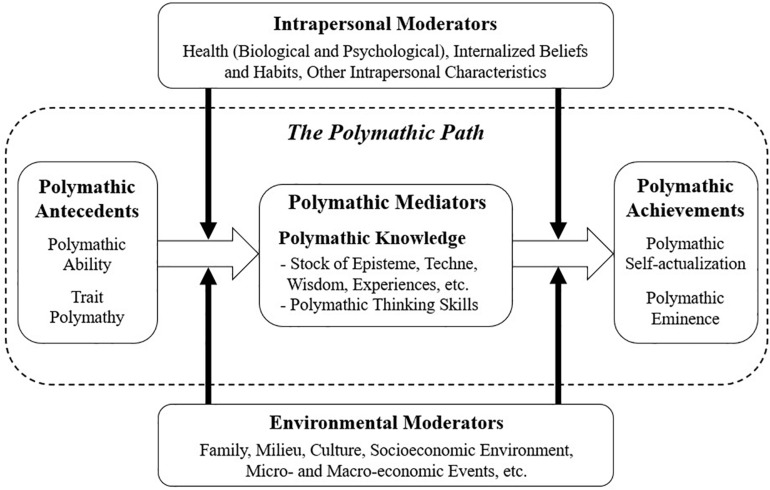
The developmental model of polymathy.

The model recognizes the existence of distinct categories of constructs that are associated with the general construct of polymathy. First, ability and trait polymathy are highly biologically based abilities and dispositions that are manifested early in life and that are propaedeutic to the acquisition and effectuation of knowledge that qualifies as polymathic. Second, polymathic knowledge involves the stock of knowledge and skills that one learns or is taught. Generally, such a stock of knowledge is qualified as polymathic when it is demonstrably both broad and profound. Importantly, this polymathic stock of knowledge should also include the learnable or developable thinking skills associated with polymathic integration (Root-Bernstein and Root-Bernstein, 2013). Finally, the polymathic achievements can refer to the portfolio of accomplishments (such as scientific articles, political ideas, innovative business products, art performances, etc.), which can be judged as polymathic by stakeholders; or it can involve the more subjective, personal goal of achievement a polymathic type of self-formation. Araki’s model also acknowledges the importance of several moderators in the path to develop polymathic knowledge or reach one’s polymathy-related goals. These moderators include intrapersonal moderators, such as internalized beliefs and habits, and environmental factors, ranging from a person’s family and milieu to major macro socioeconomic events (Figure 2).

Importantly, the model differentiates between the constructs of trait polymathy, which refers to the psychological traits and dispositions associated with polymathic behavior, and of polymathy as acquired knowledge and skills, which refers to the stock of knowledge (including thinking tactics and strategies) that one learns or is taught. This distinction will be useful in further sections, in which we take a nuanced look at both the psychological traits of polymaths and the learnable strategies that are intimately linked with the development of polymathic knowledge and which may be particularly effective in dealing with complex, uncertain, volatile and ambiguous situations, such as those set off by the COVID-19 crisis.

## Trait Polymathy and Resilience in the COVID-19 Crisis

In this section, we focus on the construct of trait polymathy and how it can contribute to resilient responses in the face of the COVID-19 crisis. Resilience is important because disruptive, large-scale crises such as the COVID-19 pandemic are likely to cause wide ranging negative effects and severe distress, leaving people and society at large with a sense of helplessness, frustration, and frailty. Although much of these events are outside people’s control, resilience is a key characteristic that enables the person to develop an active and constructive response to such events.

Given that there are currently no instruments to operationalize trait polymathy, we utilize a theoretical approach to propose that trait polymathy is associated with resilience during the COVID-19 crisis, both directly and indirectly. We begin by exploring the possible direct relationships between trait polymathy and resilience. Next, we discuss constructs pertaining to five-factor model of personality (McCrae and Costa, 1987) that are expected to have shared variance with both trait polymathy and resilience. Finally, we discuss the temperament traits, or neurochemically based individual differences (Rusalov and Trofimova, 2007), that may drive polymathic behavior and ultimately affect how one responds to conditions of great complexity and uncertainty.

### Trait Polymathy and Resilience

Traits involve stable patterns of behavior, thoughts, and emotions, and can be sometimes intertwined with values, or broad life goals that are important to people in their lives and guide their perception, judgments, and behavior (Parks-Leduc et al., 2015). Trait polymathy involves both consistent patterns of behavior and broad life goals that are intrinsecally connected with polymathy development. It is conceptualized as a multi-dimensional construct whereby each of its three dimensions mirrors a component of the general construct of polymathy. Thus, trait polymathy is composed of: (i) dispositional depth, or the dispostion to pursue depth and the high valuation of the pursuit of depth as a broad life goal; (ii) dispositional breadth, or the dispostion to pursue breadth and its high valuation as a broad life goal; and (iii) dispositional integration, or the dispostion to pursue integration and its high valuation as a broad life goal.

Resilience refers to a dynamic process that encompasses positive adaptation within the context of significant adversity (Luther et al., 2000; Oshio et al., 2018). Resilience can be examined in more detail through the constructs of ego-resiliency and trait resilience. Ego-resiliency (Block and Turula, 1963) draws on Erikson (1950/1963) suggestion that that a sense of identity and significance is required for meeting (unfavorable) life’s circumstances actively and constructively, building one’s competence for “confronting the world.” Alternatively, trait resilience focuses on personality characteristics and abilities that are specific to those who are able to successfully cope with a highly stressful life event. According to Wagnild (2009), trait resilience has five components: (i) equanimity (a balanced perspective of life and experiences); (ii) perseverance (willingness to continue the struggle to reconstruct one’s life and remain involved in the midst of adversity); (iii) self-reliance (being able to rely on one’s own strengths and capabilities); (iv) meaningfulness (realization that life has a purpose and recognition that there is something for which to live for); and (v) existential aloneness (realization that each person is unique and that while some experiences can be shared, others must be faced alone).

We propose that there are associations between the five elements of trait resilience and the three components of polymathy as a general construct. First, equanimity needs exposure to a sufficiently broad array of experiences to become functional; if the element of breadth is missing (for instance, if a person was brought up in a very closed community, and with strong censorship), the person will lack the raw materials necessary to produce effective counterpoints, thus making a balanced perspective impossible. Second, perseverance can be associated with polymathy through the channel of depth, in a similar way as the conscientiousness trait, with the addition that perseverance also relies on the integration aspect of polymathy; that is, the polymathic person remains involved in the midst of adversity through their strategic use of their network of enterprises and their correlative talents (Root-Bernstein, 1989, 2009). Third, self-reliance also relies upon a repertoire of capabilities; again, it involves the strategic use of one’s network of enterprises and correlative talents, which in turn depend on the polymathic dimensions of breadth and depth. Fourth, meaningfulness, like perseverance, rests on the person’s capacity to reach an integrative self-made worldview about one’s abilities, desires, and purposes. Finally, existential aloneness is perhaps one of the more striking convergences between polymathy and resiliency studies. As previously mentioned, people high in trait polymathy tend to develop a unique combination of profound knowledge, learning and experiences. Moreover, they refuse to be bound within extant labels in society, or to construe their identity by fitting within an existing “box.” Thus, instead of developing their identity through their belonging to a social category or group (Stets and Burke, 2000), the person high in trait polymathy tends to develop their polymathic identity via the opposite procedure: by realizing that they do not, cannot, or want not to be circumscribed within a single group or domain of knowledge and experience (Cotellessa, 2018). Thus, it is not surprising that many polymaths have reported “existential aloneness” during their interviews, even though this theme was not part of the pre-structured topics of discussion.

Research has also underscored that individuals with high ego-resiliency are resourceful in adapting to novel situations and capable of shifting their behaviors appropriately, drawing on a versatile set of cognitive and social procedures (Oshio et al., 2018). This description evokes a natural comparison with the polymathic components of breadth, depth and integration. First, the resourcefulness associated with ego-resiliency must be based on a sufficiently broad repertoire of knowledge and skills. Second, for it to work in different context, it must also be sufficiently profound. Finally, such resourcefulness must be well-integrated into a person’s self-efficacy. Moreover, highly polymathic people may have faced particularly difficult social and professional challenges due to their multiple interests that may have required a high level of ego-resiliency. This is consonant with the findings in Cotellessa (2018) in which polymaths reported facing significant adversity regarding their career choices. For example, the polymathic individuals interviewed reported finding it difficult to avoid giving “mixed messages” and building an easy-to-communicate “personal brand,” which could make them easier to hire in the job market. One positive adaptation found by some of them was to become entrepreneurs. Another adaptation was to build a set of skills that was both rare and valuable for employers, so that the polymathic person would enjoy a favorable bargaining position. Additionally, approximately half of the polymaths interviewed reported coming from dysfunctional upbringings and having few financial resources in their early life. Nevertheless, all but one of them reported finding positive adaptations from such conditions. They contended that instead of hindering their polymathy development, their lack of resources incentivized them to become broader and better learners (i.e., more polymathic) either because they could not outsource tasks to others, or because they needed to rely on their ability to generate more resources to get out of a bad situation.

Overall, the previous arguments show that polymathic individuals are expected to be resilient and particularly able to generate positive adaptations to adverse conditions; i.e., they tend to have developed the resources that help a person deal with unexpected crises in an active and constructive manner. Thus, when a major event such as the COVID-19 crisis comes in the life of a polymathic person, who had previously developed a versatile set of cognitive and social procedures for dealing with difficult life circumstances, and has probably exercised their ego-resiliency numerous times in the past, one should expect that, all other things being equal, this polymathic person will handle this situation with more adaptability and more creativity.

### Interplay With Personality Traits

Personality plays a cardinal role in resilience and previous research has proposed an association between certain general personality factors and polymathy. Particularly, three of the personality traits associated with in polymathic behavior (openness, conscientiousness and neuroticism) have been previously associated (in the same direction) with resilience in the literature (Oshio et al., 2018).

Openness to experience (McCrae and Costa, 1987) is expected to be associated with trait polymathy mainly through the components of breadth and integration. Openness involves the possession of a wider range of interests and an increased likelihood of exploring novel ideas and approaches in one’s professional and avocational spheres. People high in openness also tend to be imaginative and flexible in examining their ideas. Besides that, they are more likely to question and re-examine commonly held assumptions within a field (Kaufman et al., 2010). Relatedly, Damian (2017) and Gocłowska et al. (2018) have highlighted the role of diversifying experiences in the making of more creative (and polymathic) individuals; that is, they investigated how highly unusual and unexpected events or situations (e.g., unusual educational experiences, early life adversity) can push individuals outside the frameworks of their ordinary everyday lives, leading them to embrace new and uncommon ideas. As the person is pushed by these events, it is expected that those with a pre-existing tendency of behavior aligned with openness will extract more from those “pushes” than those with an opposite tendency. On the whole, the characteristics associated with openness as a personality trait are in line with both the theoretical descriptions of polymathic behavior in Araki (2018, 2015) and Burke (2012), and the empirical findings by Cotellessa (2018); Sriraman (2009), as well as the data, interviews and tests collected by Eiduson (1960, 1962, 1966), and later analyzed by Root-Bernstein et al. (1993, 1995, 2008) and Root-Bernstein and Root-Bernstein (2020a). In sum, given that both polymathy and openness entail the exploration of one’s interests to a large extent, and the thinking beyond artificial disciplinary boundaries (Burke, 2012; Cohen, 2015), one could expect that those commonalities would be reflected in an intimate relationship between the two constructs.

Conscientiousness is expected to be associated with trait polymathy mainly through the component of depth. One of the facets of conscientiousness involves an overarching tendency to be “prepared” (Roberts et al., 2014, p. 1317). Interestingly, “preparedness” (in the Latin form of *parata*) is a key element of polymathy since the Renaissance, appearing in the very first treaty on the subject (Wower, 1603/1665). According to Wower, polymathy requires the person being *prepared* to conduct high-quality “‘inspection or autopsy’ of unknown things” (Deitz, 1995, p. 145). This involves the progressive refinement of one’s judgment (*iudicium*) and culminates in what Wower regards as one of the noblest faculties of a polymath: the critical judgment of the written word (critical grammar) (Deitz, 1995, p. 147). Like Wower, modern scholars have also associated other conscientiousness facets, such as achievement striving, self-discipline, and competence, with polymathic behavior. For instance, Root-Bernstein (2009) highlights that the distinction between a dilettante and a polymath involves the latter being able to put a significant amount of time and effort into their interests. Likewise, both Burke (2012) and Simonton (2017) posit that polymathy requires knowledge or expertise above the superficial level. Therefore, to attain the level of knowledge and expertise associated with full-fledged polymathy, the conscientiousness-like traits of preparedness and industriousness may play a key role.

The third personality factor that can be associated (negatively) with trait polymathy is neuroticism. Neuroticism represents the tendency to experience distress and its facets include propensity toward tension, irritability, discomfort with oneself, being more easily intimidated, not resisting temptations, and not remaining calm under pressure (McCrae and Costa, 1987). The negative association with a polymathic person is expected because the development of polymathy involves a combination of going deep and broad, with intense investment in knowledge and expertise acquisition in multiple domains, which means “intruding” domains heavily defended by gatekeepers (Burke, 2012). It is expected that since a young age, the polymathic person will not only demonstrate the aptitude to navigate well in these different realities but also have had early exposure to the challenges involving polymathic behavior, necessarily leading to the development of functional coping strategies if they remain on this path. Therefore, especially the last four facets of neuroticism (discomfort with oneself, being more easily intimidated, not resisting temptations, and not remaining calm under pressure) go counter to polymathic behavior. Particularly in a society that is institutionalized toward specialism, and discourages, de-incentivizes or is outwardly hostile to polymathic behavior (Burke, 2010; Araki, 2020), neuroticism is not likely to facilitate the undertaking of the challenging polymathic path. In other words, as someone high in neuroticism faces greater challenges in becoming a polymathic person, they may end up considering a different path.

### Interplay With Temperament

Temperament refers to neurochemically based individual differences that emerge early in life and remain consistent (Trofimova et al., 2018). The functional ensemble of temperament is a model that contends that there are consistent formal-dynamical aspects of behavioral regulation that show up universally across situations and contexts, involving (i) the maintenance and endurance of chosen behaviors; (ii) the speed of integration of given behaviors; (iii) the reactivity and sensitivity to specific types of reinforcers; and (iv) emotionality. In this article, we draw on this literature to focus on four particular temperament traits are expected to be associated with trait polymathy (probabilistic processing, plasticity, mental endurance, and neuroticism) and that will ultimately affect how people high in trait polymathy respond to adverse situations and develop resilience in the face of events such as the COVID-19 crisis.

First, *probabilistic processing* is a trait regarding the behavioral orientation toward gathering a wide range of information about the frequency and causes of events, facilitating the prediction of their future occurrence (Trofimova, 2019). It involves efficient extraction and processing of new knowledge, as well as the ability to classify and operate with disparate information. Thus, it is important for the polymathic capacity to rapidly and effectively sort information, as well as to construct and refine one’s “theories,” predictions and expectations about the world. Second, *plasticity* is a trait regarding the assignment of priorities to specific sources of information or features of objects, as well as to the changing of these priorities according to individual needs (Trofimova, 2019). Thus, plasticity involves the generation of “new programs of behavior” in changing situations, and it is associated with the corrections “on the go” necessary for the rapid kind of learning described by Kaplan and Kaufman (2017). Third, *mental endurance* refers to the ability of an individual to accept new knowledge and to sustain prolonged attention and mental work, such as solving problems and performing decision-making activities (Rusalov and Trofimova, 2007). Thus, it involves the ability to sustain the amount of attention necessary to reach the optimal state of concentration conducive to profound learnings (cf. Csikszentmihalyi, 1990); it is then associated with the component of depth. Finally, regarding the aspect of emotional regulation, *neuroticism* as a temperament trait refers to the sensitivity of an individual to the probability of failure and entails the tendency to avoid novelty, unpredictable situations and uncertainty (Rusalov and Trofimova, 2007). Thus, like its counterpart in personality research, the temperament trait of neuroticism is also expected to have a negative relationship with polymathic behavior.

Overall, the study of the biological bases of behavior is a growing and promising area in individual differences and can lead to several insights in polymathy studies (see also Zeidán-Chuliá and Argou-Cardozo, 2018). By delving into the neurochemically based aspects of behavioral regulation, we can arrive at a more comprehensive picture regarding the phenomenon of polymathy, especially under the person and process perspectives in polymathy studies.

### A Note on the Important Role of Chance

It is important to discuss the role of chance in the interaction between the person and a large-scale event like the COVID-19 crisis. For instance, the effect of the COVID-19 crisis on a person can range from devastating to even positive, depending on the timing, the situation, and the external factors in which the person is embedded. Thus, while for some the COVID-19 pandemic may represent a rare opportunity to use one’s time in isolation to embrace their (multiple or not) interests, for others it will be a catastrophic event, which will severely reduce their degrees of freedom and be the cause of incommensurate emotional distress. Very importantly, research has shown that major events tend to exacerbate the so-called “Matthew effect,” in which those who are already in a privileged position will have even more abundance while those who do not have much, will “lose even the little that they have.” For instance, Pluchino et al. (2018) demonstrated, through an agent-based model, that (un)lucky events can be more determinant regarding a person’s success than their level of raw talent. Consistent with that, Simonton (1979) had previously modeled how scientists “hit” their successes and his findings also indicated support for a “chance theory” instead of a “genius theory” in which talent would make the biggest difference. Still, even in Pluchino et al. (2018)’s model, there is still a role for personal characteristics. For instance, in their model, “talent” is what allows the person to exploit lucky opportunities, and also moderates this relationship.

## Push-Type Polymathy: Learning Polymathic Strategies to Adapt in the COVID-19 Crisis

For dealing with a disruptive crisis such as the COVID-19 pandemic, knowledge is a decisive asset. Thus, we have argued that one’s disposition to pursue breadth, depth and integration of knowledge, as well as the development of an actual polymathic “repertoire” of knowledge and thinking skills can be determinant for one’s success and well-being when facing significant adversity. In this section, our focus shifts from those individuals that are “naturally” polymathic (i.e., those high in trait polymathy) to focus on how conditions that are adverse, uncertain and complex can elicit behaviors that are intimately associated with polymathy, and which can be developed by individuals regardless of their level of trait polymathy. For this, we draw a parallel between the “push” and “pull” types of motivation to advance the idea of a “push” type of polymathic behavior; that is, when reasons unrelated to their personal attributes put people in a position that “pushes” them toward the development of a more polymathic (broader, deeper and more integrated) set of knowledge and skills.

We contend that in these situations, individuals of the most diverse intellectual profiles will find much benefit in acting in a more polymathic way; that is, they will be in a better position by acquiring and integrating ideas, methods, concepts, principles, techniques, or materials from domains different than those they are used to, and by using them to generate novel and effective solutions. The COVID-19 crisis is a foremost example of an “exogenous shock,” with an extremely adverse, uncertain and complex nature, which we theorize that will demand the development of coping behaviors that are similar to the polymathic behaviors described in previous sections. Thus, the COVID-19 crisis will “push” individuals toward greater expansion and integration of new knowledge in order to create novel and useful solutions for them and those around them during the crisis.

A very illustrative example of how the COVID-19 crisis can act in pushing the expansion in knowledge is found in businesses owners. Entrepreneurs, especially in smaller business, are already expected to be more balanced in their investment strategy regarding knowledge and skills (Lazear, 2004). This should not come as a surprise, especially for owners of small businesses. Given that they are not able to outsource many activities, they must integrate tasks much beyond the usual scope of a corporate business leader; that is, small entrepreneurs often have to learn how to be the marketers, accountants, controllers, operational workers, and even cleaners of their own businesses. Nevertheless, besides these traditional demands, the COVID-19 crisis has provided many businesses with a new litmus test: which businesses will stay afloat? Which businesses will remain useful and relevant for people? And perhaps most crucially: which organizations can learn, and adapt their business model, to fit the new world in which we now live? Thus, to deal effectively with the crisis, many owners had to develop their polymathic knowledge to new heights. In the next paragraphs, we will utilize the case of a daycare center in the northeastern United States to illustrate how the quick obtention of a broader range of knowledge and skills allowed this owner to keep her business afloat—a business in a sector severely affected by the COVID-19 crisis.

### The Case of a Day Care Provider

In mid-March of 2020, the daycare temporarily closed due to the pandemic. By mid-July, there were plans put into place for a re-opening. From analyzing these plans, it became clear that the owner of this small business stretched her knowledge boundaries: she did a lot of research, sought input from others, and devised a robust plan for the reopening of the daycare center, which would disrupt most—if not all—previous school procedures.

The plans included the following: parents are not allowed to go into the building anymore. Instead, parents drive up close to the facility in their vehicles. Each vehicle must have the child’s name and teacher’s name posted on a piece of paper, showing through the car window. A daycare representative comes to the vehicle, takes the child’s temperature with a calibrated, infrared thermometer, and if the temperature is below 100 degrees Fahrenheit, the child is retrieved with his or her belongings and go into the daycare center. Upon entering the center, the child removes their shoes, washes hands, and places their individual, labeled water bottle which parents would have prepared into a specified, individual location for that child. Communal water fountains are not used anymore. In the classroom, headcounts were lowered, and classes were rearranged to promote individual play, which required a novel pedagogical plan. Room dividers, shelving, and cones are now used to indicate physical boundaries. Individual supply bags are also provided to each child. Classroom windows are now left open whenever feasible. The outdoor playground will be used more frequently to allow for fresh air and additional spacing. The same teachers will take care of the same class throughout the day, to reduce exposure. Presumably for financial reasons, business hours were also adjusted, shortened by about one hour.

Moreover, the daycare made plans for how to explain important new concepts to the children, such as “social distancing,” “physical boundaries” and “personal boundaries,” as well as their own terms like “helicopter arms” to help children understand and act according to the new standards. Additionally, the daycare designed activities to not require close physical contact between the kids. They used items like pool noodles, hula-hoops, taped areas, and wall markings to encourage children to maintain space from others, eliminating large-group activities. Finally, all staff have been trained in proper procedures to reduce the spread of infectious disease, and also in the procedures for how to report cases of COVID-19. Sick children will be isolated immediately and must be picked up right away. To return to the daycare center, they must be symptom-free or cleared with a doctor’s permission.

In sum, this is an example of a small business owner learning about many different aspects of how to operate her facility that she never had to contend with before. For instance, she had to learn about disease prevention. She had to think about the business finances while also figuring out ways to support social distancing. She had to figure out how to adjust meal time, cleaning and sanitation procedures, group size and ratios, staff safety considerations, staff training, how to handle illnesses and report them, and also how to create a video tutorial further explaining what the new procedures would look like in order to maximize compliance. This business adapted: the owner learned about a variety of elements as explained above, which probably were not her areas of expertise, to modify her business model. In sum, this is a business exemplar that has so far survived the pandemic. The use of flexibility, openness, learning, and information sharing were the keys to this business’s success in the context of COVID-19 crisis.

Overall, we posed that the development of creative polymathy can occur through multiple channels. However, polymathy must always involve the acquisition of learnings, experience and expertise in terms of breadth, depth and their integration into novel and useful solutions for the self or for others. We also posited that one important differentiation concerned the direction of these learnings; that is, if they are internally motivated or externally motivated. We referred to the former as pull-type polymathy and to the latter as push-type polymathy. The pull-type polymathy is in fact one instance of purposeful design in learning; that is, when individuals decide to expose themselves to deliberately enhance and enrich their experiences and capacities. All these approaches have in common the fact that the learner is the protagonist, guided by their own curiosity and exerting control of learning process (Guglielmino et al., 1987). Conversely, the push-type polymathy can be associated with incidental learning, which refers to learning that is unintentional and may arise from the unplanned exposure to a significant and unexpected event (Marsick and Watkins, 2001). That is, by just living life, individuals are bound to be exposed to a variety of events that may trigger different types of thinking, behaviors, ideas, etc., and such learning can happen in various ways: through observation, socializing with other people, or solving problems. To exemplify this second type of polymathic learning, we described how the owner of a small business was pushed to acquire a new set of learnings and skills due to the unplanned event of the COVID-19 pandemic.

### Creative Polymathy and the Different Types of Problems

For the last 100 years, the standard approach in Western society to solve problems has been that of increasing specialization and departmentalization (Burke, 2012). This approach has indeed led to astonishing technological and scientific progress; however, the more “VUCA” (volatile, uncertain, complex, ambiguous; Bennis and Nanus, 1985) our problems and society becomes, the less effective the segregationist approach is (Cohen, 2015; Gombrich, 2016; Araki, 2020).

The COVID-19 crisis is a foremost example of a multidisciplinary VUCA problem whose solution requires the highest grade of creativity. Although the pandemic itself can be seen as primarily a public health crisis, it has also led to an unprecedented economic and social crises, and because of complex interdependencies that the COVID-19 crisis entails, even a good effort to solve one aspect of this problem may create or exacerbate other problems. For instance, although public health measures such as isolation, social distancing, and quarantine are being implemented to combat the spread COVID-19, the isolation and loss of social contact have also been associated with psychological symptoms such as depression and anxiety, which in turn increases the risk of suicide (Dsouza et al., 2020; Gunnell et al., 2020).

**FIGURE 3:**
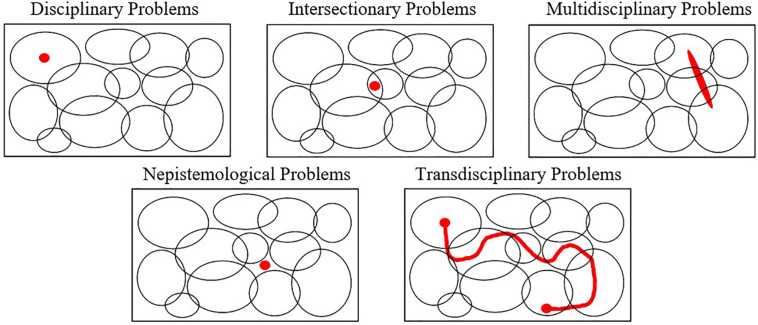
Five categories of problems according to their disciplinary boundaries.

To understand more systematically the strengths and pitfalls of our current approach and to better identify new possible ways of thinking to solve “wicked,” VUCA problems, it pays to examine with greater precision the way that different problems can (or cannot) be circumscribed within the current disciplinary organizations. Thus, we draw on previous studies (Root-Bernstein, 1982, 1989, 2003; Gombrich, 2016) to advance a typology with five categories of problems concerning their disciplinary boundaries: (i) Disciplinary Problems; (ii) Intersectionary Problems; (iii) Multidisciplinary Problems; (iv) Nepistemological problems; and (v) Transdisciplinary Problems (Figure 3).

First, disciplinary problems are those that fit nicely within the current set of facts, concepts, techniques, heuristics, themes, questions, goals, and criteria that are employed by a single discipline. Most of the problems that students are asked to solve in high school or their undergraduate programs are disciplinary problems. There is generally one (and rarely a few more) technique within the current knowledge in the discipline that perfectly solves the problem.

Second, intersectionary problems are those that involve facts, concepts, techniques, heuristics, themes, questions, goals, and criteria that are shared by two or more disciplines. Thus, these problems can be solved by different disciplinary angles. However, this problem occurs in an already expected and “stylized” interdisciplinary space, such as “biochemistry” occurs at the intersection of biology and chemistry, but very importantly, these problems can be still treated with the traditional toolkit of one or both disciplines.

Third, multidisciplinary problems involve three or more disciplines. They are complex and often systemic—that is, it cannot be treated locally, with the facts, concepts, techniques, heuristics, themes, questions, goals, and criteria of a single discipline. These problems are increasingly more challenging than the previous two and require coordination of integration between disciplines but are still amenable to a combined disciplinary approach.

Fourth, nepistemological problems are those that, for some reason, fall outside the set of facts, concepts, techniques, heuristics, themes, questions, goals, and criteria covered by any extant discipline; that is, they are hidden problems, the “unknown unknowns,” and the “blank spots on the map of human knowledge” (Root-Bernstein, 2003). Because they have been off the radar, when these problems do emerge, they will inherently challenge the validity of the current paradigms and boundaries of knowledge (Kuhn, 1962).

Fifth and finally, transdisciplinary problems are the combination of multidisciplinary and nepistemological problems. That is, not only they involve multiple areas of knowledge but also the blank spots between those areas. The COVID-19 crisis is an exemplar of a transdisciplinary problem. Thus, because the wicked nature (Rittel and Webber, 1973) of these problems—involving volatile conditions, and a large degree of complexity, ambiguity, and uncertainty—they pose a difficult challenge to coordination efforts and to leaders in different domains (Araki, 2015)^1^.

### Polymathic Coping Ability for the COVID-19 Crisis

Given the different categories of problems described above, as well as the different approaches to the acquisition, processing and utilization of knowledge that entail polymathic thinking and disciplinary thinking, we propose that there are different optimal points of learning between polymaths and specialists regarding the level of the “VUCAness” of the situation. To illustrate this, we utilize two curves in a two-dimension plot, denoting the optimal psychological performance for the polymath and the specialist.

**FIGURE 4:**
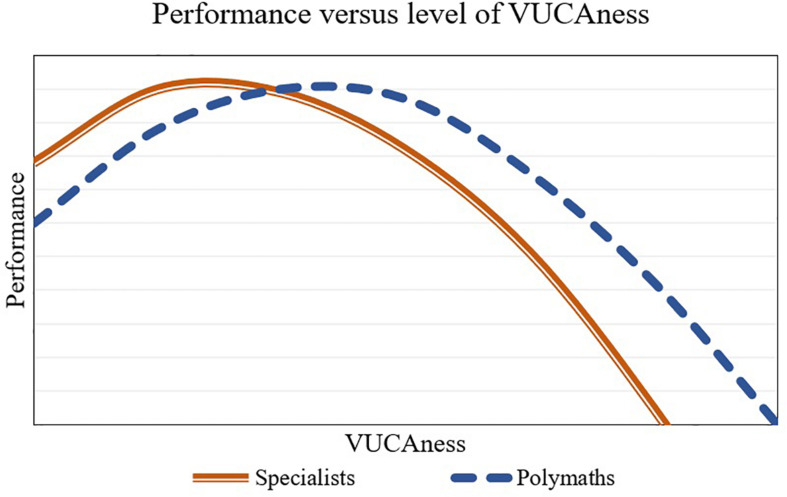
Performance of polymaths and specialists in different levels of VUCAness.

We define the specialist as the person whose breadth of expertise (profound knowledge) does not significantly depart from the typical sets of knowledge in the field. In contrast, the polymath is a person who has profound knowledge, skills and expertise that covers significantly different domains, and cannot be well circumscribed within a typical disciplinary boundary. The horizontal axis in Figure 4 represents the degree of “VUCAness;” that is, how much volatility, uncertainty, complexity and ambiguity is involved in the situation at hand. Finally, the vertical axis represents the coping ability; that is, the capacity to utilize one’s own conscious effort to solve personal and interpersonal problems in a stressful environment. In this paper, coping also refers to the capacity to retain learning performance under stressors stemming from the VUCAness of the problem environment.

We propose that polymathic people tend to display greater coping skills and that they can better retain learning performance under VUCA stressors. In addition, we propose that the performance of polymaths peak at a greater level of VUCAness than that of the specialists. To explain why this might be the case, we draw on the literature on arousal systems under deterministic and non-deterministic situations (Trofimova and Robbins, 2016). This research strand poses that the cortical and subcortical networks involved with more deterministic, well-defined, or explicit (concrete) features of situations are different from those involved with non-deterministic (probabilistic) contextual processing and the resolving of uncertainty utilizing implicit (abstract) features of events. Three of the four temperament traits associated with trait polymathy are those related with monoamine systems modulating the arousal for or maintenance of those non-deterministic properties of behavior: probabilistic processing; plasticity and mental endurance. Interestingly, the fourth temperament trait associated with polymathy, neuroticism, involves a negative emotional reactivity to uncertainty; thus, all of the four temperament traits associated with high trait polymathy have to deal with how one behaves under uncertainty.

Compared to the polymathic person, we pose that the specialist will achieve optimal experience and peak performance with tasks that involve a smaller degree of novelty and VUCAness. Additionally, we propose that their performance will be less sensitive to boredom than that of the polymath. That is, specialists will tend to be more content performing the tasks that are somewhat repetitive, or which they have already mastered. Thus, even in tasks with less variation and novelty, their sustained attention is expected to work satisfactorily—albeit not at the peak level.

Conversely, polymaths will achieve optimal experience and peak performance at a later point in the graph (Figure 4). That is, they need more novelty and more VUCAness to feel challenged and to really engage in the task. Unfortunately for them, most tasks in life may not be so interesting and, because of that, such tasks might be perceived as not worthy of the polymath’s full attention. Therefore, while performing these “easy” tasks, polymaths tend to become bored, easily distracted, and even neglectful. Because of this, their performance tends to be worse than those who are more content in sustaining more repetitive or deterministic behaviors, in which there is little novelty.

Finally, when the level of VUCAness starts growing too much, the bounded rationality kicks in and it becomes increasingly unwieldy and ineffective to try to make sense of and process so much information with our naturally limited attention. However, we posit that polymaths are comparatively better at stretching their absorptive capacities, not only in terms of new knowledge assimilation but also in terms of the utilization of their thinking tools, to the limit. Additionally, they arguably have more experience dealing with VUCA situations, since they are expected to have been exposed to greater novelty and diversity of situations and environments in the past. Because of this, polymathic individuals may have had more opportunities to learn how to adjust their behavior rapidly and effectively to complex, changing contexts.

### Combining the Forces of the Polymath and the Specialist

The complexity leadership theory (Uhl-Bien et al., 2007) argues that the more complex a problem is the more likely it will require complex, dynamic adaptations. If this is true, then the complex, wicked problems stemming from the COVID-19 crisis demand something different from our dominant approach today. The disciplinary type of thinking that thrived in the 19th and early 20th century is not conducive to deal with problems that insist on not being well-circumscribed within the standard disciplinary boundaries or which requires exploration of new possibilities instead of exploitation of known techniques, facts, methods, etc.

When society, organizations and institutions favor, incentivize, or only allow for the approach of the narrow specialist, we become more vulnerable to wicked transdisciplinary problems such as the COVID-19 crisis. We pose that the current siloed, segregationist approach to thinking is a model that served very well during and in the aftermath of the industrial revolution, but which is now showing its limitations in an increasingly clear manner. By discouraging polymathic behavior, society is also discouraging people from following their self-directed learning styles; that is, from learning in ways that diverge from the standardized ways that most people feel compelled—or are obliged—to follow, and to create their own learning opportunities based off of their curiosity. By constraining learners and knowledge workers to the convention and typicality of a limited set of disciplinary facts, concepts, techniques, heuristics, themes, questions, goals, and criteria, we are limiting our ability to generate novel, useful and surprising solutions. In an environment when the great majority of field members have been exposed to largely the same set of ideas, the polymath has a great advantage both in terms of possessing “items of knowledge” that are distinct from the crowd and of having had the possibility to develop their own unique “thinking skills.” Such uniqueness in knowledge and thinking has in fact been reported as an important source of the polymath’s creative advantage (Root-Bernstein et al., 1995; Root-Bernstein et al., 2008).

We expect that in both the industry and the academia of the future, polymaths should play a critical role in coordination with deep specialists. In fact, this should go beyond collaboration, understood as exchange of information. It should entail a combination of vertical and transversal integration aiming at a superordinate synthesis. That is, different strands—from the most fundamental to the most applied, encompassing disciplinary angles from the most typical to the most unusual but potentially useful—should be analyzed, analogized, abstracted, scrutinized, and synthesized in order to allow for a comprehensive understand of the problem, its nature and the possible solutions. This would capitalize on the key features of both polymathic and disciplinary thinking. On the one hand, polymathic thinking is extremely conducive to the generation of creative ideas and utterly necessary for dealing with the “wicked” transdisciplinary problems. On the other hand, disciplinary thinking has, for centuries, allowed not only for the organization of a messy, fuzzy reality into manageable “blocks,” but also to the understanding of incredibly complex things and phenomena by breaking them down into smaller parts and making them more amenable to investigation. Thus, together, the specialists and the polymaths can make the best combined use of the breadth, depth and integration of knowledge—old and new—allowing for better bridges across disciplines, better coordination and synthesis, and moving toward more innovation. In sum, for the best of outcomes, both approaches must coexist in a synergistic manner, within a culture that fosters and intelligently capitalizes on the particularities of each type of thinking.

## Conclusion: Toward a More Polymathic World?

What lessons can be derived from the COVID-19 crisis? We have argued in this article that humanity will benefit from a more polymathic approach, especially in the context of major crises, such as COVID-19. However, the current way our society operates is not conducive to developing and supporting polymathic thinking; we are still clinging to the age of specialization, in which the dominant paradigm is for everyone to be a specialist, focused in a narrow easy-to-communicate area. And, particularly important, in the age of massive information, those polymaths who give “mixed messages” will have more difficulty in getting attention, recognition, in being appreciated for their talents, and may even struggle to find a job. Such societal arrangement is not only reductive but also dangerous, especially in the context of the kinds of problems humanity now faces. Therefore, we need a collective culture shift to better ready ourselves for future crises—a culture shift that involves greater appreciation for the polymathic approach, both at the individual and collective levels.

At present, the segregationist approach leaves a great deal of potential polymathic power untapped. For instance, academia largely works with siloed departments and fragmented curricula, making it hard for faculty and students to tackle systemic problems in a more comprehensive or integrative manner (Cohen, 2015). This reality is also found in most businesses and government organizations: problems that by any chance fall into a gap between departments or cross departments that do not usually work together will have no one responsible for it, and tend to be surprisingly hard to solve in this kind of structure—even if they are simple problems. Additionally, a very unfortunate facet of the segregationist worldview has reared its head in the popular and political circles, with the over-simplistic split of “health versus economy” in the COVID-19 crisis discussion as a prime example. Again, this divisive approach puts society as a whole in a more vulnerable position by driving people away from potentially useful connections that could arise from new syntheses, combinations, or integration. Thus, although it is not surprising that transdisciplinary problems require an approach that goes beyond the segregationist or disciplinary thinking, our culture has not reached this point yet. Given the value of the polymathic approach, and given that this value is not being recognized by the dominant models that are currently guiding behaviors and creating incentives in society; thus, there is an urgent need to change the pattern of such collective behaviors and assumptions, which are so intertwined with our way of perceiving, thinking and feeling the world. In other words, it is necessary to create an environment that is more conducive to scientific and cultural evolution based on the pillars of depth, breadth and integration.

In conclusion, humanity has received a proverbial “wake up call” that our complex world problems demand a more polymathic approach. The COVID-19 crisis is demanding solutions that involve more collaboration across industries and sectors, as well as an approximation among countless parties. The path for better readiness in the future for major disasters like the COVID-19 crisis entails the creation of an environment and culture where people can be rewarded for taking a polymathic perspective. And it must be true to all sorts of segments, including academia, industry, government, non-profit organizations, etc. Institutionally, if we want to solve wicked, VUCA problems faster and more efficiently, it is vital to engage in a intelligent approach that can add both breadth and integration to the extant fragmented expertise. This novel approach would entail all aspects traditionally associated with polymathy: looking at the intersection and gaps between disciplines, applying lessons and strategies from one domain to another, looking for the fundamental connections, and seeing the bigger picture while others may be so focused on a reduced part of the reality. Thus, if polymathy is allowed to flourish in our culture, it will bring vitality, adaptability and agility to our organizations and institutions. More polymathy means that people will bring more of their true self to their exchanges with society, without the need of hiding their “disparate” avocations, or concurrent or past vocations in other areas because they are afraid to send a “mixed message” and gatekeepers will not even consider them. We are complex beings, but our institutions are currently not up to that complexity. If this cultural change toward acceptance of polymathy occurs, society will unleash the power of polymathy and we, as a whole, will be in a much more advantageous position to deal not only with the present crisis, but with the great challenges of the uncertain future.

## Data Availability Statement

The original contributions presented in the study are included in the article/supplementary material, further inquiries can be directed to the corresponding author/s.

## Author Contributions

MA had the initial idea for this conceptual-theoretical article. MA and AC wrote the article together. Both authors contributed to the article and approved the submitted version.

## Conflict of Interest

The authors declare that the research was conducted in the absence of any commercial or financial relationships that could be construed as a potential conflict of interest.

## Footnotes

1 This figure was inspired by previous works from David Staley, Robert and Michele Root-Bernstein, Peter Burke, Dean K. Simonton, and Richard Feynman.
